# Multi-Stage Particle Separation based on Microstructure Filtration and Dielectrophoresis

**DOI:** 10.3390/mi10020103

**Published:** 2019-01-31

**Authors:** Danfen Yin, Xiaoling Zhang, Xianwei Han, Jun Yang, Ning Hu

**Affiliations:** Key Laboratory of Biorheological Science and Technology, Chongqing University, Ministry of Education, Bioengineering College, Chongqing University, Chongqing 400030, China; yindf@cqu.edu.cn (D.Y.); zhangxiaoling@cqu.edu.cn (X.Z.); 20161901019@cqu.edu.cn (X.H.); bioyangjun@cqu.edu.cn (J.Y.)

**Keywords:** Microfilter, Dielectrophoresis, Particle separation, micropillar

## Abstract

Particle separation is important in chemical and biomedical analysis. Among all particle separation approaches, microstructure filtration which based particles size difference has turned into one of the most commonly methods. By controlling the movement of particles, dielectrophoresis has also been widely adopted in particle separation. This work presents a microfluidic device which combines the advantages of microfilters and dielectrophoresis to separate micro-particles and cells. A three-dimensional (3D) model was developed to calculate the distributions of the electric field gradient at the two filter stages. Polystyrene particles with three different sizes were separated by micropillar array structure by applying a 35-Vpp AC voltage at 10 KHz. The blocked particles were pushed off the filters under the negative dielectrophoretic force and drag force. A mixture of Haematococcus pluvialis cells and Bracteacoccus engadinensis cells with different sizes were also successfully separated by this device, which proved that the device can separate both biological samples and polystyrene particles.

## 1. Introduction

Microfluidic technology involves the control and manipulation of small amount of fluid confined in micron-sized geometry [1,2]. Microfluidic operations have many advantages, including faster analyses, minimum consumption of samples and reagents, shorter reaction times, and high-throughput screenings [3]. Moreover, miniaturization makes it possible to develop portable devices, which means that miniaturized laboratory equipment can be taken where it is needed. The influence of microfluidic technology in the academic community has rapidly increased over the last ten years because it has a number of potential applications in such areas as biological analysis [4], clinical examination [5], and food safety inspection [6,7].

Particle separation is important in chemical and biomedical analysis [7], and microfluidic techniques have been effective at separating particles. Many microfluidic methods have been developed to separate particles [8] using the flow field, microstructure, or forces created by electricity [9], optics [10,11,12,13], acoustics [14,15], magnetics [16,17,18], hydrodynamics [9,19,20], or gravity [20,21]. Some of these methods require fluorescent immunolabeling or magnetic labeling of the targeted or non-targeted particles, which is not only complicated but also possibly pollutes the reactants [22]. Other methods do not require label and separate particles by their intrinsic qualities, such as dielectric properties, deformability, size, deterministic lateral displacement, etc. 

Microfilters, which are frequently used in many fields for tasks such as particle capture, enrichment and separation, have a controllable pore size and distribution. Microfilters do not need sophisticated injection systems, which make them efficient, fast, and simple.

Wilding et al. [23] demonstrated a micropillar array structure to separate blood cells and particles from nanoliter samples. However, the use of micropillar array structures to separate cells can lead to blockages in the flow path, limiting the development of the structure. Recently, different kinds of microfluidic chips were developed to avoid blockages, such as independent micropillars based filter, etc. Mohamed et al. [24] designed a sorting chip consisting of four channel segments. The channel’s width sequentially contracts from 20 μm to 15 μm, then to 10 μm, and ends at 5 μm, while the height remains constant. Cells injected into the chip were captured at different segments according to their sizes. Tan et al. [25] proposed another filtering structure to separate cancer cells. On the microfluidic device, each filter element consists of three independent micropillars arranged in a circular arc with a 5 μm gap, which ensured that only larger cancer cells cannot pass and other cells can bypass from the side due to the increased flow resistance. McFaul et al. [26] developed a funnel-shaped micropillar array with a large gap in upstream and a small gap in downstream to reduce the influence of blockages. These methods can alleviate the degree of clogging to a certain extent, but they cannot completely prevent blockages.

Dielectrophoresis (DEP) is also a useful and simple technology for particle separation [27]. Positive dielectrophoresis (pDEP) and negative dielectrophoresis (nDEP) are both used to separate blood cells, tumor cells (including CTCs), algae cells, etc. The DEP force is a net force caused by the non-uniform electric field around the particles, which could be generated by the geometry of the electrodes or insulators [28]. The most frequently used DEP approaches are electrode-based DEP and insulator-based DEP [8,29,30]. The electric field gradient in iDEP is produced by using insulator micropillars, which in eDEP is produced by complex shaped electrodes. Particle separation using iDEP can avoid many problems that may occur in eDEP. Mohammadi et al. [31] developed an efficient micropost array to capture particles using insulator-based dielectrophoretic generated by a DC voltage source, and did numerical simulation to find the most efficient design of the post array, which demonstrated the effectiveness of the combination of micropost array and dielectrophoresis 

However, the blockage problem frequently occurs in particle separation by using microfilter devices. Recently, our group developed a new microfluidic device made of a series of filters to separate particles using AC voltages. AC voltages were chosen to generate iDEP because the electrodes in AC voltages were less likely to be electrolyzed than in the DC voltages. Compared with the proposed methods, our device combined the advantages of microfilters and dielectrophoresis. Under the manipulation of dielectrophoretic force, the blocked particles were pushed off from filters to ensure that the particles can be separated continuously. This device can separate particles of three or more different sizes simultaneously, by adding more separation stages. More importantly, all filter stages can work at a constant frequency and voltage, by adjusting the geometry parameters of micropillars and ITO electrodes.

## 2. Materials and Methods

### 2.1. Related Theories

The DEP technology is an electrokinetic transport mechanism driven by polarization [32] that could be a useful tool to control the motion of particles. When surrounded by an electric field, homogeneous dielectric particles will be polarized [33,34] and the time-averaged DEP force can be expressed as [35,36]:

(1) FDEP=2πεma3Re[K(ω)]∇|Erms|2

where *ε_m_* is the permittivity of the medium, *a* is the particle radius, ∇∣*E_rms_*∣^2^ is the gradient of the square of the RMS electric field, and *K*(*ω*) is the Clausius–Mossotti factor. For a particular sphere, the real part of *K*(*ω*) ranges from −0.5 to 1, and is determined by the frequency of the applied field and the complex permittivity of the medium [37]. *K*(*ω*) can be calculated by:

(2) $$K(\omega )=\frac{{\tilde{\varepsilon }}_{p}-{\tilde{\varepsilon }}_{m}}{{\tilde{\varepsilon }}_{p}+2{\tilde{\varepsilon }}_{m}}$$

where *ω* is the angular field frequency, ${\tilde{\varepsilon }}_{p}$ is the complex permittivity of the particle and ${\tilde{\varepsilon }}_{m}$ is the complex permittivity of the medium. For isotropic homogeneous dielectrics [38], the complex permittivity can be expressed as:

(3) $$\tilde{\varepsilon }=\varepsilon -j\frac{\sigma }{\omega }$$

with *j* = $\sqrt{-1}$, *ε* and σ being the is permittivity and conductivity, respectively.

*F*_DEP_ is balanced with the drag force of particles in the fluid [39]. For a homogeneous spherical particle in a laminar flow regime, the Stokes drag force can be expressed as:

(4) $$F_{drag}=6\pi \eta av$$

where *η* is the fluid viscosity, *a* is the radius of the particle, and *v* is the velocity of the particle relative to the fluid. 

### 2.2. Experiment

#### 2.2.1. Microfabrication

**Figure 1:**
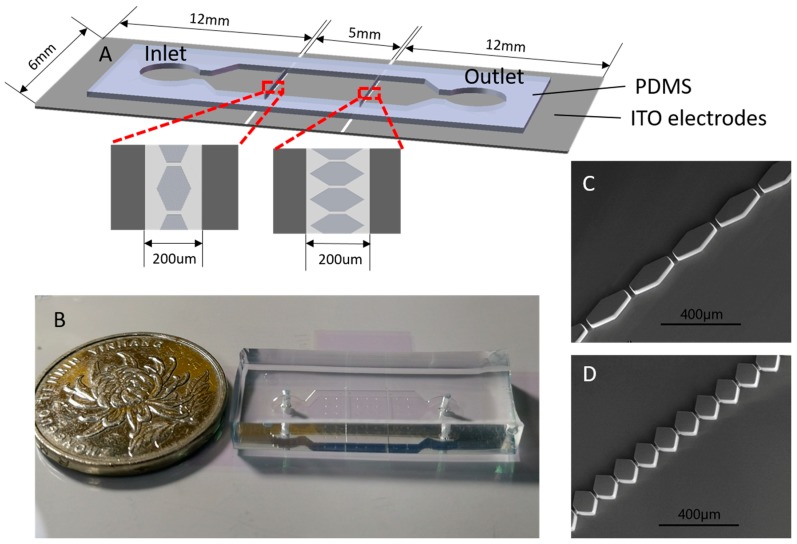
(**A**) Schematic illustration of the microfilter device, the distance of the two ITO electrodes is 200 μm; (**B**) a picture of the microfilter device; (**C**,**D**) scanning electron micrograph of micropillar structures with a height of 50 μm, and the gap of the micropillars are 25 μm and 14 μm, respectively.

The microfilter device in the experiment was fabricated using the standard soft lithography technique [22,40,41]. To produce a 50 μm thickness structure, a positive mold was fabricated by using SU-8 3050 (Microchem, Westborough, MA, USA) spin-coated on a 3-inch silicon wafer (ePAK, Austin, TX, USA) at 3000 rpm for 30 s [42]. A mold was realized by using a photolithography aligner device (URE-2000/25, Institute of Optics and Electronics, Chengdu, China). Replicas of the mold were made in polydimethylsiloxane (PDMS, Dow Corning, Midland, MI, USA). As shown in Figure 1A, the microfluidic filter device had two reservoirs with a diameter of 4-mm and a long micro-channel with a length of 2.3 cm, a width of 6 mm, and a depth of 50 μm. Considering the convenience and reliability of connection between the signal generator and ITO electrodes, a 30 mm × 6 mm ITO glass was chosen to fabricate ITO electrodes. In addition, to integrate more separation stages and induced a high strength DEP force under a lower voltage, the distance between electrodes should be miniaturized. Finally, the distance between two ITO electrodes was 200 μm, which was wider than the micropillars (shown in Figure 1A). The filter consisted of a series of hexagonal micropillars (Figure 1C,D), which had two interval sizes inside the microchannel (25 μm and 14 μm). The injection port was fabricated using a 3-mm diameter puncher and the port to plug outlet tubing was fabricated using a 1-mm diameter puncher. 

For better observation, indium-tin-oxide (ITO) [43] (220-nm ITO film thickness, 7 Ω/sq.) was used to fabricate the electrodes due to its good transparency. A bare ITO electrode should be carefully washed by using acetone, isopropanol, ethanol, and ultrapure water in turns before use. Then the ITO electrode was fabricated with a SU-8 2000 series negative resin using the standard soft lithography technology. Finally, the PDMS replica was sealed onto the ITO glass with electrodes via O_2_-plasma activation (PDC-MG, Chengdu, China) of both surfaces. 

#### 2.2.2. Experimental Solutions

Polystyrene particles were suspended in a 1-mM phosphate-buffered saline (PBS) buffer with a low electrical conductivity of 0.17 S/m to minimize Joule heating in the filter region [8,44,45]. Polystyrene particles of 37-μm, 16.3-μm, and 9.7-μm diameters were suspended in the PBS buffer at a concentration of 10^4^−10^5^ particles per milliliter. To avoid the adhesion of particles to the channel walls and minimize the interactions between particles, Tween 20 (TP1379, Bomeibio, Hefei, China) was added to the mixture solution at a concentration of 0.1% *v/v* [41]. 

Haematococcus pluvialis (FACHB, Wuhan, China) and Bracteacoccus engadinensis (FACHB, Wuhan, China) were cultured in Blue-Green Medium (BG11, FACHB, Wuhan, China). A mixture of Haematococcus pluvialis and Bracteacoccus engadinensis were diluted in the culture medium at a concentration of 10^4^−10^5^ cells per milliliter, and 0.4 g/mL sorbitol was added to suspend the cells.

#### 2.2.3. Experimental Manipulation and Visualization

**Figure 2:**
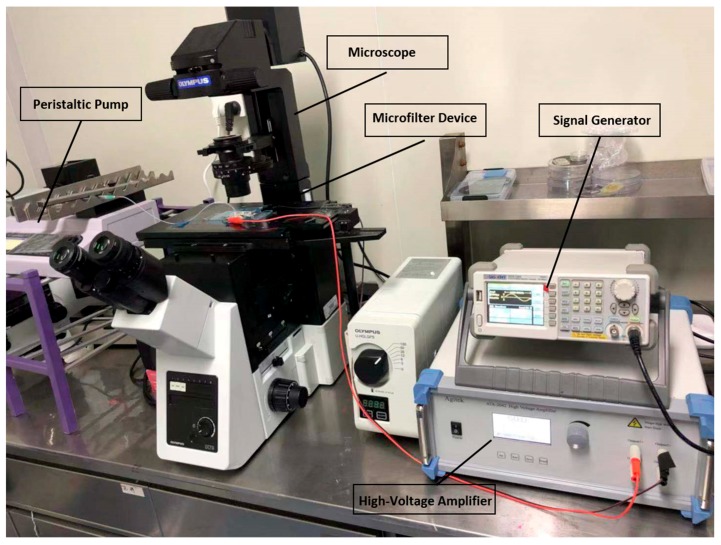
Experimental setup including the microfilter device, microscope, peristaltic pump, signal generator, and high-voltage amplifier.

A pipe connecting the outlet and the peristaltic pump was inserted into the outlet reservoir. The particle solution was added in the inlet reservoir by a pipette and introduced into the micro-channel by suction provided by the peristaltic pump. Serious leakage may occur during solution injection, but suction can help avoid this disadvantage because of the excessive pressure. Before the experiment, the microfluidic filter device was washed with 1-mM PBS buffer without particles for 5 min [46]. The inlet reservoir was brimmed with the particle mixture solution using a 100-μL pipette when the experiment began. As shown in the Figure 2, the sinusoidal signal used in the experiment was supplied by a function generator (SDG1020, Siglent, Solon, OH, USA) and a high-voltage amplifier (ATA-2042, Agitek, Xi’an, China) [41]. The AC electric field was fixed at 10 KHz and 35 V peak-to-peak value during polystyrene particles experiments. For algal cells, the AC electric field was fixed at 8 KHz and 100 V peak-to-peak value [47,48]. A microscope (IX73, Olympus, Tokyo, Japan) was used to monitor particle motion, and a digital single lens reflex (Canon, Tokyo, Japan) was used to record videos and images in the microfluidic filter device through the microscope. 

## 3. Results and Discussion

### 3.1. Electric Field Gradient Distribution

Clausius-Mossotti factor value will determine whether the particles were subjected to positive dielectrophoresis (pDEP) or negative dielectrophoresis (nDEP). It depends on the parameters of particles, buffer solution, and applied electric signal. In our separation system, 1mM phosphate buffer was used as a buffer solution. The conductivity σm and permittivity of buffer solution are 0.17 S/m and 7.04 × 10^−10^ F/m, respectively. And the conductivity of polystyrene particles could be calculated by *σ_p_* = 2*K_s_*/*r* (r: the radius of the particle). The recommended value of *K_s_* for surface conductance is 10^−9^ S. The permittivity of polystyrene particles is 2.04 × 10^−10^ F/m. Clausius–Mossotti factor could be calculated by Equation (2) and Equation (3). The results showed that the value was always negative in the frequency range of 0–10^7^ Hz. In addition, considering the ITO electrodes are easier for electrolysis at a frequency less than 10 KHz and the same DEP force can be induced by a lower voltage and higher frequency electric signal, 10 KHz was selected as the operating frequency.

**Figure 3:**
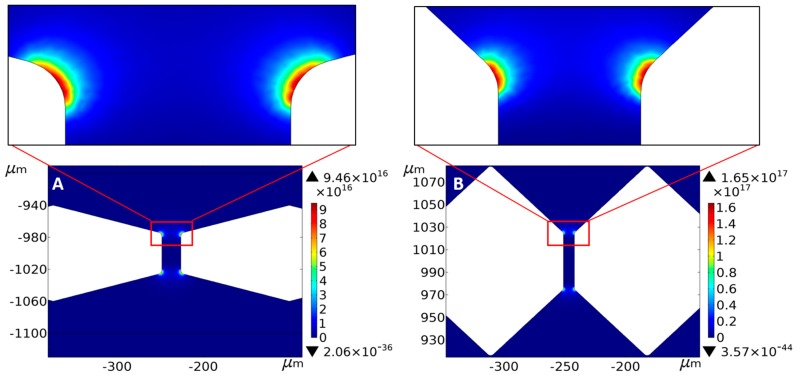
Distributions of ∇∣*E_rms_*∣^2^ near the two stages microfilters (**A**,**B**), when 35 Vpp at 10 KHz is applied.

To find a proper structure of the filter to separate particles of three sizes in two stages of filters by applying the same voltage, we developed a three-dimensional (3D) model in COMSOL Multiphysics 5.0 (COMSOL, Newton, MA, USA) to investigate the distributions of the electric field gradient and intensity. Figure 3 illustrates the distributions of the electric field gradient at the two stages. The strongest intensity is 9.46 × 10^16^ V^2^/m^3^ in Figure 3A and 1.65 × 10^17^ V^2^/m^3^ in Figure 3B. The electric field gradient at the second stage must be higher than that at the first stage because the DEP force is proportional to the cube of the particle radius (*a*^3^). 

The non-uniform electric field is generated by applying an AC electric field of 35 Vpp and 10 KHz using ITO electrodes placed as shown in Figure 1A [49,50]. The strongest intensity and gradients of the electric field exist near the edge of filter. The particle mixture was injected into the inlet well and flew through the two-stages filter in sequence due to pressure-driven flow. When entering into the non-uniform electric field region, the trajectories of some particles change because of the nDEP force. When the particles passed the first stage filter, 37-μm particles moved to the opposite direction of the flow because of the strong DEP force, while 16.3-μm and 9.7-μm particles passed through the first stage filter. In the second stage filter, 16.3-μm particles were trapped before the second stage filter, while 9.7-μm particles passed through the second stage filter.

### 3.2. Separation of Three Different Particles

For particular particles and media, changing the AC frequency influenced the direction of the DEP force. A frequency of 10 KHz was chosen to ensure the particles experienced a negative DEP force. During the experiment, we found that if the flow velocity was fixed, the filters were easily clogged at low AC voltages; however, a strong DEP force generated at high AC voltages, which prevented all particles from passing through the first stage filter. Similarly, if the AC voltage was fixed, a high velocity caused congestion, while a low velocity influenced the separation efficiency. Thus, there is a balance between voltage and fluid velocity [51]. To achieve better continuous microfiltration, voltage and velocity should be optimized. Experiments were conducted with AC voltages ranging from 30−50 Vpp and flow velocities ranging from 0.5−2 μL/min [51]. The best separation condition for the particles was found to be 35 Vpp and 1 μL/min, where in the first stage the 16.3-μm and 9.7-μm particles can pass through and in the second stage the 9.7-μm particles can pass through the filter; none of them got trapped when the voltage was above 35 Vpp.

**Figure 4:**
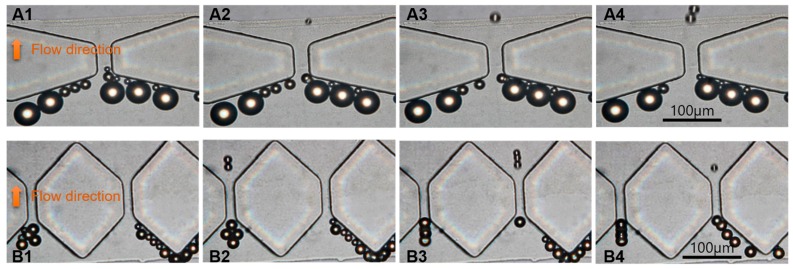
The separation process of 37-μm, 16.3-μm and 9.7-μm particles. (**A1**–**A4**) The first stage. (**B1**–**B4**) The second stage.

Figure 4 shows a continuous separation of 37-μm, 16.3-μm, and 9.7-μm polystyrene particles in the microchannel (The complete separation process is recorded in Video S1 and Video S2). The AC voltage and frequency imposed were 35 Vpp and 10 KHz, respectively, and the flow velocity was 1 μL/min. As seen in Figure 4A1–A4, in the first stage, only the 16.3-μm and 9.7-μm particles could pass through the filter, while 37-μm particles were stopped by the filter (25-μm interval). Figure 4B1–B4 show results near the second stage; the filter (14 μm interval) stopped the 16.3-μm particles and pushed them to the roof of hexagon and only allows the 9.7-μm particles to pass through. It can be seen from Figure 4B1–B4 that not all 9.7-μm particles can pass through the second stage filter at once. Some particles were bunched to form a pearl chain and suddenly pass through the filter as a group. 

The microfilter would be blocked right after the 50-μL particle mixture is pumped into the micro-channel without applying AC field [51], which limits the popularization and application of microstructure filtration methods. Figure 4 demonstrate that the device kept working when the AC field (35 Vpp, 10 KHz) was applied, indicating the microfluidic device solved the blockage problem successfully, which makes continuous separation possible. 

### 3.3. Separation of Algae Cells

Haematococcus pluvialis and Bracteacoccus engadinensis were also used to verify the feasibility of the device. Haematococcus pluvialis is famous for its high content of astaxanthin, which is the strongest antioxidant in nature and plays an important role in aquaculture, health care, and cosmetics industries [52]. Haematococcus pluvialis cells are often mixed with other algae cells in nature, thus, it is necessary to sort and purify Haematococcus pluvialis. Bracteacoccus engadinensis cells were mixed with Haematococcus pluvialis cells to mimic the natural condition. 

**Figure 5:**
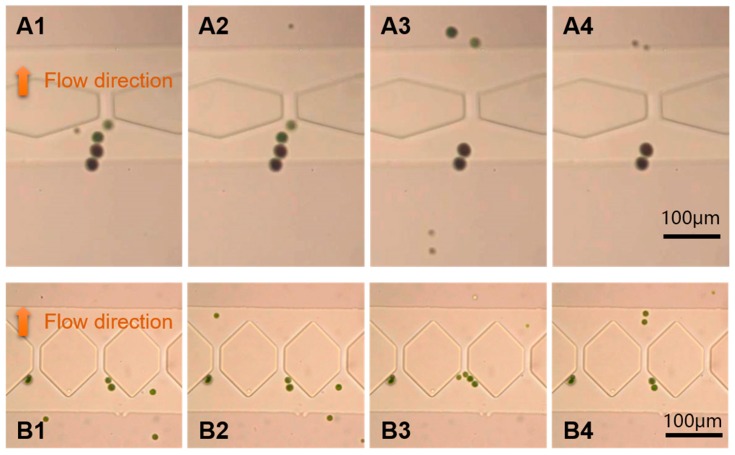
Separation process of Haematococcus pluvialis cells and Bracteacoccus engadinensis cells at a voltage amplitude of 100 V and a frequency of 8 KHz. (**A1**–**A4**) The first stage. (**B1**–**B4**) The second stage.

The sizes of Haematococcus pluvialis cells are between 15 and 30 μm, and the sizes of Bracteacoccus engadinensis cells are 10 to 15 μm. In this study, we applied an AC signal with a voltage of 100 V and a frequency of 8 KHz, and the flow velocity is the same as the particle separation. As shown in Figure 5A1–A4, all of the Bracteacoccus engadinensis cells and some of the smaller Haematococcus pluvialis cells can pass through the filter unimpededly in first stage with a pore size of 25 μm, but larger Haematococcus pluvialis cells larger than 25 μm cannot pass. Like the 37-um particles, large Haematococcus pluvialis cells were pushed away by the negative dielectrophoretic force near the entrance of the first stage filter. The results of the second stage are depicted in Figure 5B1–B4. Smaller Bracteacoccus engadinensis cells can pass through the filter with the size of 14 μm, while Haematococcus pluvialis cells and large Bracteacoccus engadinensis cells were trapped before the filter under dielectrophoretic force. The complete separation process is recorded in Video S3 and Video S4.

Due to large size, only a few Haematococcus pluvialis cells can pass the first stage since more Haematococcus pluvialis cells can been seen before the entrance of the first stage filter (Figure 5A1–A4), while only a few Haematococcus pluvialis cells can be seen in the second stage (Figure 5B1–B4). Accordingly, because of the small size, all of the Bracteacoccus engadinensis cells can pass through the first stage, and most of them can pass through the second stage and reach the outlet directly. After separation, there are only Haematococcus pluvialis cells left in the channel before the first stage, Bracteacoccus engadinensis cells in the outlet, and merely a small amount of these two algae cells with similar sizes in the channel between the first stage and the second stage. Thus different sizes of Haematococcus pluvialis cells and Bracteacoccus engadinensis cells were separated.

## 4. Conclusions

We presented a microfluidic filter device that combines the advantages of negative dielectrophoretic force and microfilters, to separate particles of different sizes. Microfilter is one of the most widely used particle/cell separation methods due to simple operation. However, blockages limit its popularization and applications. We were committed to solving the blockage problem of microfilter and purify Haematococcus pluvialis cells using the simplest filter structure in this study. A 3D model was developed to analyze electric field distribution. Based on the simulation results and particles size, micropillars based separation device was designed and fabricated. In addition, the geometry parameters of micropillars were optimized to ensure two separation stages work by using a generator. The feasibility of this method was demonstrated by the continuous flow separation of polystyrene particles with three different sizes. Haematococcus pluvialis cells and Bracteacoccus engadinensis cells were also separated in this device without blockages phenomenon.

Considering our device is based on the filtration theory, this microfluidic device could separate complex sample, which contains many kinds of (bio)particles with different size, by integrating several separation stage with appropriate micropillars and applying appropriate AC signal. By optimizing the geometry parameter of micropillars, and adjusting the distance between two adjacent micropillars, circulating tumor cells, white blood cells, red blood cells, or blood plasma could be separated from whole blood samples [53,54,55].

The developed microfluidic filter device can be conveniently fabricated and generates a strong dielectrophoretic force near the filter of each stage. The device can separate particles of different sizes efficiently with minimal Joule heating due to the use of pressure-driven flow and AC electric field. However, some particles, which should theoretically pass through the filter, were trapped due to the too strong DEP force and adhesion between particles. It reduces the separation efficiency [6]. Additionally, (bio) particles collection structures should be integrated, to avoid too much (bio) particles block the flow path. 

## Supplementary Materials

The following are available online at http://www.mdpi.com/2072-666X/10/2/103/s1, Video S1: The separation process of particles in the first stage, Video S2: The separation process of particles in the second stage, Video S3: The separation process of Haematococcus pluvialis cells and Bracteacoccus engadinensis cells in the first stage, Video S4: The separation process of Haematococcus pluvialis cells and Bracteacoccus engadinensis cells in the second stage.
